# Impact of COVID-19 on Cancer Care Delivery in Africa: A Cross-Sectional Survey of Oncology Providers in Africa

**DOI:** 10.1200/GO.20.00569

**Published:** 2021-03-10

**Authors:** Yehoda M. Martei, Tara J. Rick, Temidayo Fadelu, Mohammed S. Ezzi, Nazik Hammad, Nasreen S. Quadri, Belmira Rodrigues, Hannah Simonds, Surbhi Grover, Luca Incrocci, Verna Vanderpuye

**Affiliations:** ^1^ Department of Medicine (Hematology-Oncology), University of Pennsylvania, Philadelphia, PA; ^2^ Department of Radiation Oncology, Erasmus Medical Center, Rotterdam, the Netherlands; ^3^ Dana-Farber Cancer Institute, Boston, MA; ^4^ University of Nairobi, Nairobi, Kenya; ^5^ Queen's University Cancer Center of Southeastern Ontario, Kingston Health Science Center, Kingston, ON, Canada; ^6^ Allina Health, St Paul, MN; ^7^ African Organization for Research and Training in Cancer, Cape Town, South Africa; ^8^ Division of Radiation Oncology, Medical Imaging and Clinical Oncology, Tygerberg Academic Hospital, Cape Town, South Africa; ^9^ Department of Radiation Oncology, University of Pennsylvania, Philadelphia, PA; ^10^ National Center for Radiotherapy and Nuclear Medicine, Korle-Bu Teaching Hospital, Accra, Ghana

## Abstract

**PURPOSE:**

The COVID-19 pandemic has disrupted cancer care globally. There are limited data of its impact in Africa. This study aims to characterize COVID-19 response strategies and impact of COVID-19 on cancer care and explore misconceptions in Africa.

**METHODS:**

We conducted a web-based cross-sectional survey of oncology providers in Africa between June and August 2020. Descriptive statistics and comparative analysis by income groups were performed.

**RESULTS:**

One hundred twenty-two participants initiated the survey, of which 79 respondents from 18 African countries contributed data. Ninety-four percent (66 of 70) reported country mitigation and suppression strategies, similar across income groups. Unique strategies included courier service and drones for delivery of cancer medications (9 of 70 and 6 of 70, respectively). Most cancer centers remained open, but > 75% providers reported a decrease in patient volume. Not previously reported is the fear of infectivity leading to staff shortages and decrease in patient volumes. Approximately one third reported modifications of all cancer treatment modalities, resulting in treatment delays. A majority of participants reported ≤ 25 confirmed cases (44 of 68, 64%) and ≤ 5 deaths because of COVID-19 (26 of 45, 58%) among patients with cancer. Common misconceptions were that Africans were less susceptible to the virus (53 of 70, 75.7%) and decreased transmission of the virus in the African heat (44 of 70, 62.9%).

**CONCLUSION:**

Few COVID-19 cases and deaths were reported among patients with cancer. However, disruptions and delays in cancer care because of the pandemic were noted. The pandemic has inspired tailored innovative solutions in clinical care delivery for patients with cancer, which may serve as a blueprint for expanding care and preparing for future pandemics. Ongoing public education should address COVID-19 misconceptions. The results may not be generalizable to the entire African continent because of the small sample size.

## INTRODUCTION

Global incidence of COVID-19 has surpassed 112 million cases and 2.4 million deaths.^1^ Patients with cancer have a higher incidence of COVID-19 and are at higher risk of severe complications and death.^2-10^ Emerging data show that the COVID-19 pandemic and control strategies have resulted in disruption of cancer care and research globally.^11-16^ Subsequently, international oncology organizations in predominantly high-income countries have developed guidelines for mitigating risk for COVID-19 infection in patients with cancer and many of these countries have developed innovative models for safe care delivery to minimize the disruption in cancer treatment.^17-19^ The implementation and impact of these guidelines on clinical outcomes remain unknown.

**CONTEXT**

**Key Objective**
How has the COVID-19 pandemic affected cancer care delivery in Africa?
**Knowledge Generated**
The pandemic response includes widespread implementation of common mitigation and suppression strategies in the African countries represented in the study. COVID-19 cancer cases and associated deaths were fairly low. However, our data highlighted disruptions and delays in care of all cancer treatment modalities. Not previously reported is the fear of infectivity leading to staff shortages and decrease in patient volumes. Our data also provide insight into how the pandemic has inspired innovative solutions in clinical care delivery for patients with cancer in Africa.
**Relevance**
Although COVID-19 cases and deaths in patients with cancer are low, reported disruptions in cancer care are concerning for potentially long-term adverse sequelae for patients with cancer currently undergoing treatment in Africa. These data provide critical lessons for innovations in care delivery to ensure continuity of cancer care during this pandemic and future outbreaks.

The African continent is now confronting an increasing number of COVID-19 cases, but relatively fewer deaths because of COVID-19 based on available data.^1^ Similarly, many African countries have employed variations of mitigation and suppression models adopted in high-income countries and those recommended by the WHO.^20^ These have been developed to avert a healthcare system onslaught of COVID-19 cases given the fragile healthcare infrastructure with relative lack of intensive care unit facilities, ventilators, and healthcare personnel.^21^ However, critical real-world data on the extent of implementation in Africa are limited. Additionally, data are lacking on specific strategies for reducing risk of COVID-19 for patients with cancer in Africa who already have a disproportionate burden of morbidity and mortality.^22^ These data are critical for understanding the impact of the pandemic on cancer care delivery for patients on the continent and for understanding strategies that were successfully implemented and well-tailored to the continuous care delivery within the complex healthcare systems of specific African countries.

The aims of this study are to characterize the scope of general COVID-19 and cancer care–specific preparedness strategies employed in Africa during the COVID-19 pandemic and to capture clinician perspectives of the impact of the pandemic on cancer care in their institutions. This study included an exploratory aim to identify myths and misconceptions that oncology healthcare providers are confronting.

## METHODS

### Study Population

The study population included any cancer care provider based in Africa, regardless of subspecialty. This included medical doctors, nurses, and allied healthcare professionals who actively provide clinical care to patients with cancer. Survey responses were collected anonymously, and participants only provided their e-mail addresses if they were interested in being contacted for follow-up studies. The research study was granted exemption by the Institutional Review Board of the University of Pennsylvania.

### Survey Design and Distribution

The web-based cross-sectional study was administered using Research Electronic Data Capture (REDCap). The domains captured in the survey included participant demographics and practice characteristics, national and institutional COVID-19 control strategies, impact of COVID-19 on care delivery, estimates of COVID-19 cancer cases and deaths among patients with cancer, and myths and conceptions encountered by providers. The survey items were drafted and finalized with multidisciplinary input from the study investigators. The survey was translated to French and Portuguese. The final survey took approximately 15 minutes to complete.

The survey was distributed using two primary methods: (1) The African Organization for Research and Training in Cancer (AORTIC) Listserv and other professional societies with focus on cancer in Africa and (2) participants were invited to share the survey link via social media platforms with other oncology healthcare workers in Africa through a snowball sampling method. The AORTIC Listserv has contact information for members in 40 countries across Africa. The survey included instructions to limit responses to one per participant. The survey was distributed from June 23 to August 14, 2020. Reminders were sent 2 weeks before the close of the survey.

### Data Analysis

Descriptive statistics were used to summarize the data on demographics and COVID-19 response strategies and impact. Comparative analysis grouped by World Bank income status was conducted. Data entries for multiple cancer centers were compared for accuracy and discrepancies and were reported in our results. All statistical analyses were carried out in SPSS Statistics version 24 (IBM Corp, Armonk, NY).

## RESULTS

### Participant and Practice Characteristics

**FIG 1:**
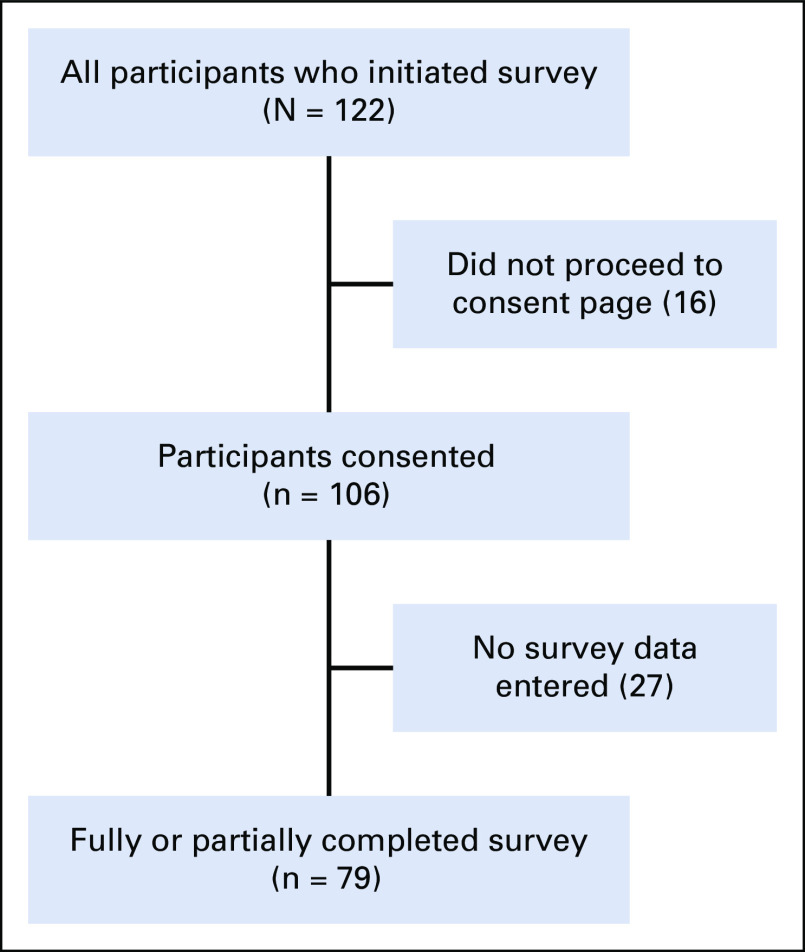
Flow diagram of survey participants.

**FIG 2:**
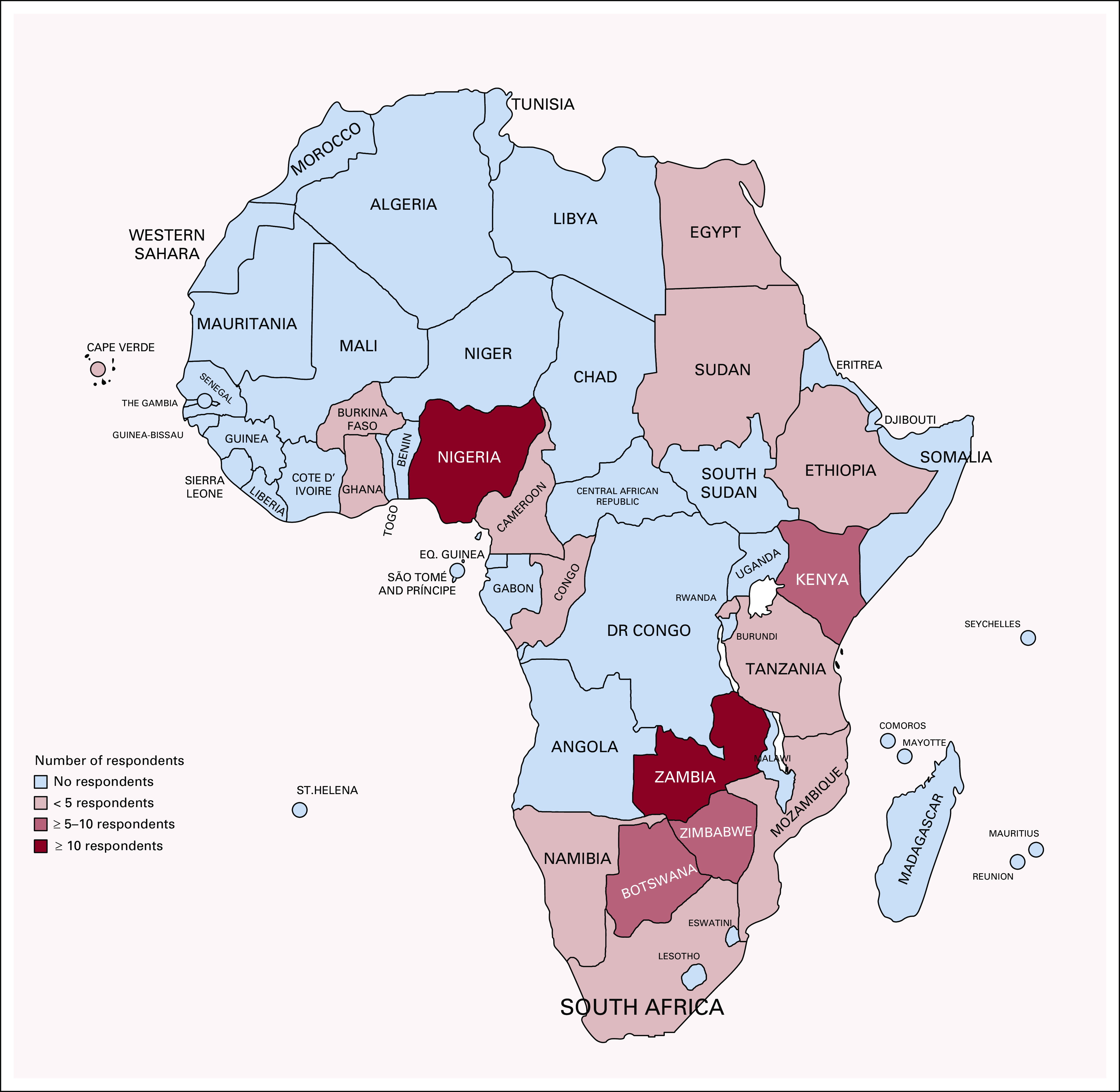
Distribution of participants across Africa.

One hundred twenty-two cancer care providers initiated the survey, of which 16 (13.1%) were excluded for not proceeding to the consent page and 27 (22.1%) consented participants excluded for not entering any data (Fig 1). There were 79 survey participants from 23 centers in 18 countries in Africa who provided partial or complete data included in these analyses (Table 1 and Fig 2). We were unable to calculate a survey response rate because of the distribution and sampling techniques employed in this study. Eleven participants did not provide the name of their institution. The countries with the most participants practiced in Zambia and Nigeria, and the majority of participants were from lower-middle-income countries. A majority of survey participants practiced in government hospitals and/or academic centers. Additional demographic and practice data are provided in Table 1.

**TABLE 1:**
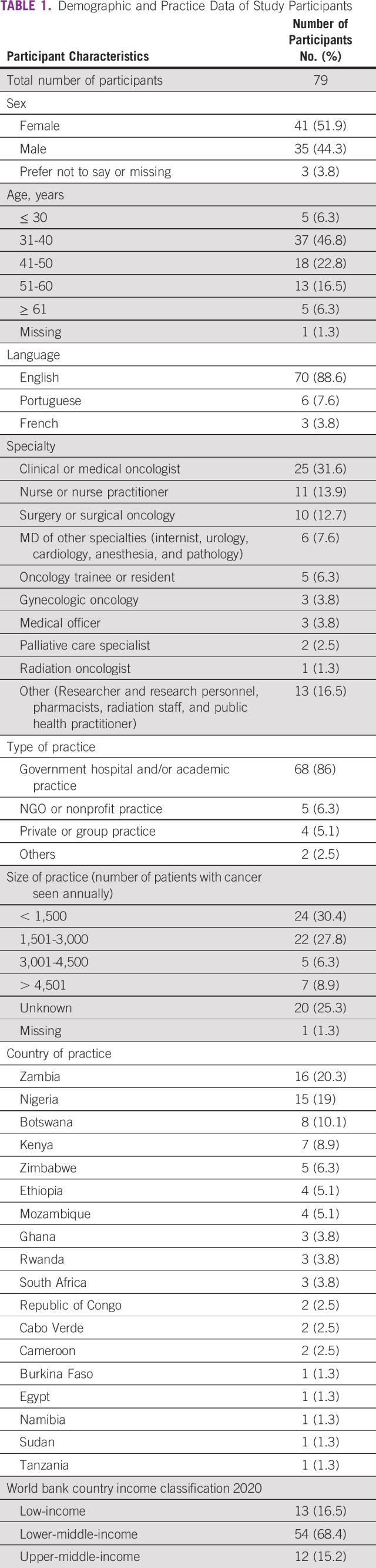
Demographic and Practice Data of Study Participants

The response strategies provided by participants are presented below and organized using classifications suggested by the Inter-Agency Standing Committee where applicable: Unless otherwise reported, there were no significant differences in the responses by income groups.^20^

### Government-Level Strategies for General COVID-19 Control

#### Mobilize all sectors and communities.

Public education was implemented by 17 African countries for which data on national COVID-19 strategy were provided. On a scale of 0-100 (with 100 being most prepared), the mean score for country preparedness rated by 61 respondents was 48.02 (median = 50).

#### Prevent, suppress, and slow transmission.

**FIG 3:**
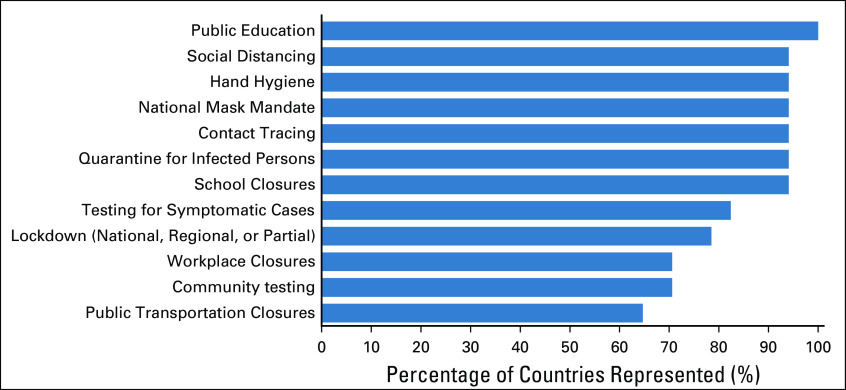
Frequency of nonpharmacologic interventions implemented for COVID-19 control in Africa. Data from 70 respondents practicing in 17 African countries.

Almost all countries (94.1%) implemented widely endorsed nonpharmacologic mitigation and suppression strategies, including social distancing, hand hygiene, national mask mandates, contact tracing, and quarantine of infected persons (Fig 3). Other interventions including lockdowns, public transportation closures, nationwide school and workplace closures, and community and symptomatic testing are summarized in Figure 3.

#### Institution-level strategies for oncology-focused COVID-19 control.

A majority of respondents reported the use of specific cancer care guidelines during the pandemic (50 of 70, 71.4%). These were primarily institution-based protocols (39 of 70, 55.7%) and/or specific guidelines from the national Ministry of Health (34 of 70, 48.6%). Few respondents reported the use of international guidelines such as European Society for Medical Oncology and ASCO, by their respective institutions (19 of 70, 27.1%).

Table 2 lists specific patient-facing and provider-facing strategies employed by cancer centers. Most common interventions reported by > 90% of respondents were temperature screening, mask mandates and social distancing for patients, and mask mandates for providers. Institution of compulsory mask mandates for patients was significantly highest in upper-middle-income countries (100% [12 of 12] of respondents), compared with 81% (44 of 54) in lower-middle-income countries and 69% (9 of 13) in low-income countries (*P* = .03). Additionally, there were a higher number of staff shortages reported in low-income (77%, 10 of 13) compared with lower-middle- (56%, 30 of 54) and upper-middle-income countries (33%, 4 of 12, *P* = .03). Survey respondents who noted the use of virtual tumor boards and/or video-based telehealth rated this favorably, with only 2 of 37 respondents reporting that it rarely worked. Internet connectivity was cited as the biggest challenge to virtual platforms (11 of 70, 15.7%). On a scale of 0-100, the mean score for institution preparedness rated by 65 respondents was 46.43 (median = 50).

**TABLE 2:**
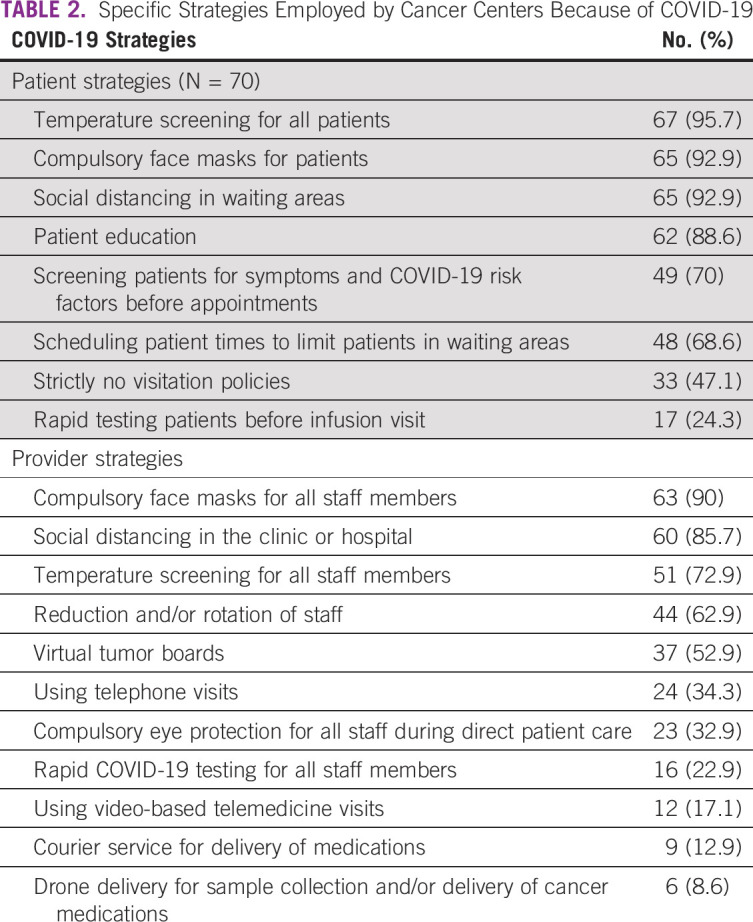
Specific Strategies Employed by Cancer Centers Because of COVID-19

#### Innovative strategies in response to the COVID-19 pandemic.

Unique strategies employed by some facilities during the pandemic include courier service for delivery of medications in Congo, Egypt, Kenya, Nigeria, Rwanda, and drone delivery for sample collection and/or delivery of cancer medications in Kenya, Rwanda, and Zambia (Table 2).

### Impact of COVID-19 on Cancer Care

#### Cancer care delivery disruptions because of the COVID-19 pandemic.

Most cancer centers remained open during the pandemic but implemented several therapy modifications (Table 3). A majority of respondents reported that patient surveillance visits for their institutions were postponed, and 30% reported that new patients experienced delayed initiation of treatment. A majority of the 21 respondents who reported delay in new patient visits in their facilities stated that patients were delayed ≤ 2 months (13 of 21, 62%). Approximately one third of respondents reported chemotherapy, radiation therapy, and surgical plan modification because of the pandemic (Table 3). A majority of modifications instituted were delays—for example, delay or withholding of palliative chemotherapy, delaying adjuvant therapy, delaying curative and radiation therapy, or delaying surgery for patients with low risk of progression (Table 3). Additional modifications reported were an increase in the use of hypofractionated and/or ultrafractionated radiotherapy and modification of palliative care treatment plans, including decreased inpatient hospice referrals (Table 3). Low-income countries were significantly more likely to delay curative radiation therapy compared with lower-middle- and upper-middle-income countries (4 of 13, 0 of 54, and 0 of 12 respectively; *P* < .001). A detailed description of the therapy modifications for the different treatment modalities in patients with cancer is provided in Table 3. Disruptions in cancer care also included interruption of research activities in 35 of 47 participants involved in research (74.5%).

**TABLE 3:**
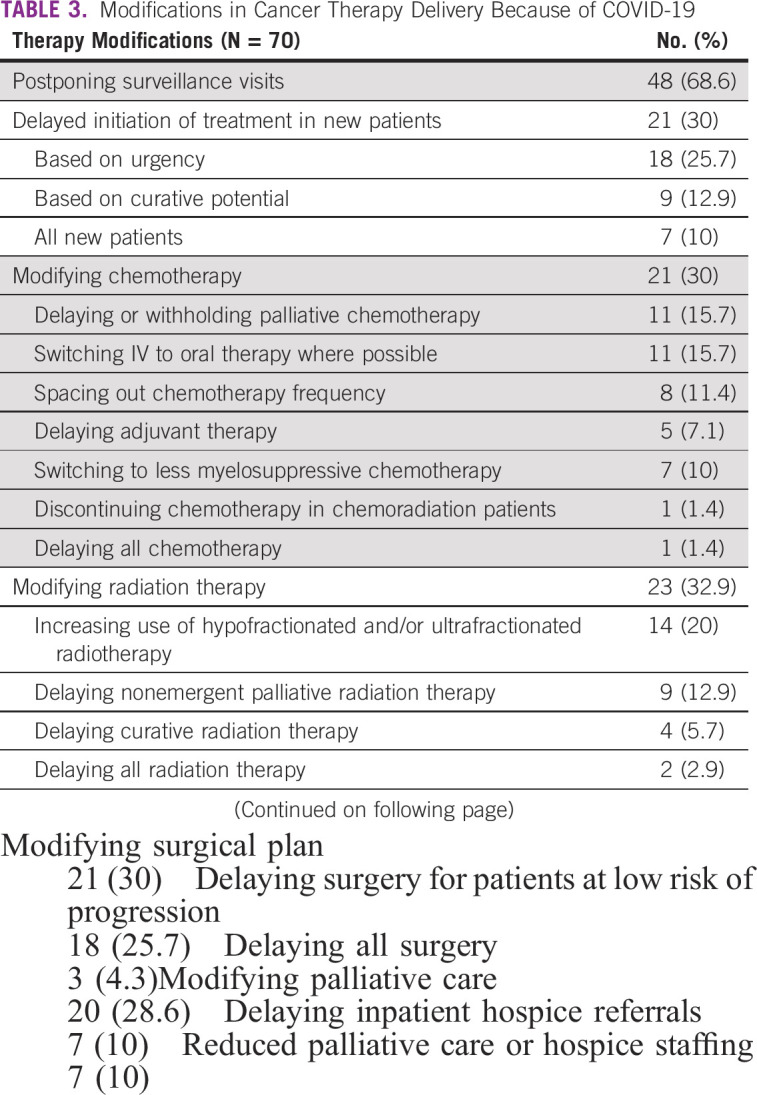
Modifications in Cancer Therapy Delivery Because of COVID-19

More than three quarters of respondents reported that their centers had a reduction in patient volumes, because of patient factors (fear of exposure and economic or financial barriers), national-level factors (travel restrictions implemented during the lockdown), and institutional-level factors (facility-initiated reduced in-person visits and implementation of remote or telemedicine visits by cancer care facilities) (Table 4).

**TABLE 4:**
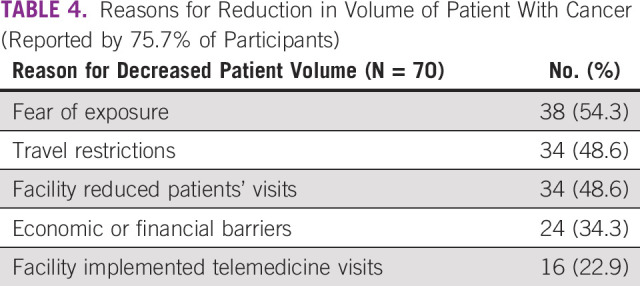
Reasons for Reduction in Volume of Patient With Cancer (Reported by 75.7% of Participants)

A majority of respondents reported staff and personal protective equipment (PPE) shortages at cancer centers (Table 5). The frequency of the reasons for staff shortages, including self-isolation and fear of contracting the virus being the topmost reasons, is listed in Table 5. A majority of participants reported that they had access to surgical masks and gloves. However, only 48.6% (34 of 70) had access to N95 masks and goggles and/or face shields, gowns, or powered air–purifying respirator or controlled air purifying respirator (1 of 70, 1.4%). 30% (21 of 70) reported access to cloth masks, and 11% (8 of 70) were obligated to provide their own PPE. Other resource shortages reported during the pandemic were cancer therapeutics and analgesics shortages (Table 5).

**TABLE 5:**
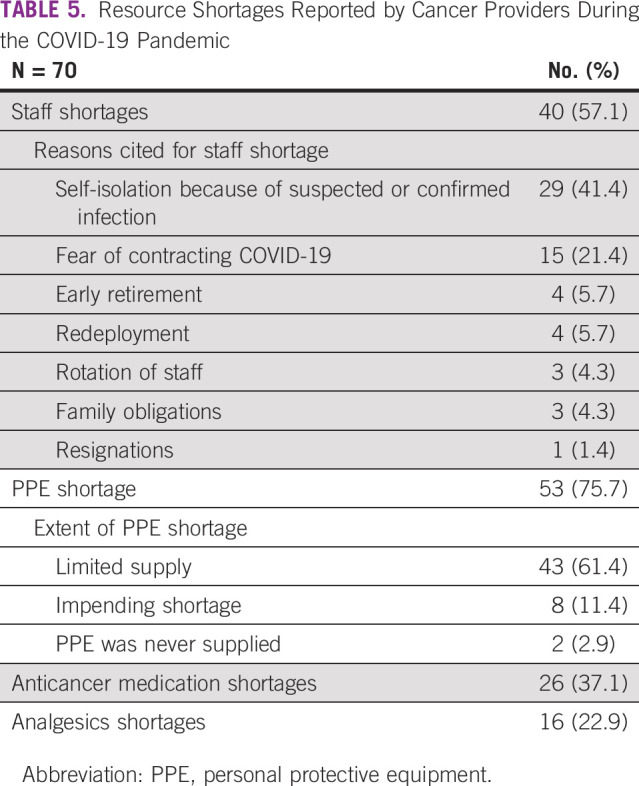
Resource Shortages Reported by Cancer Providers During the COVID-19 Pandemic

#### Reported COVID-19 cases and deaths in patients with cancer.

**FIG 4:**
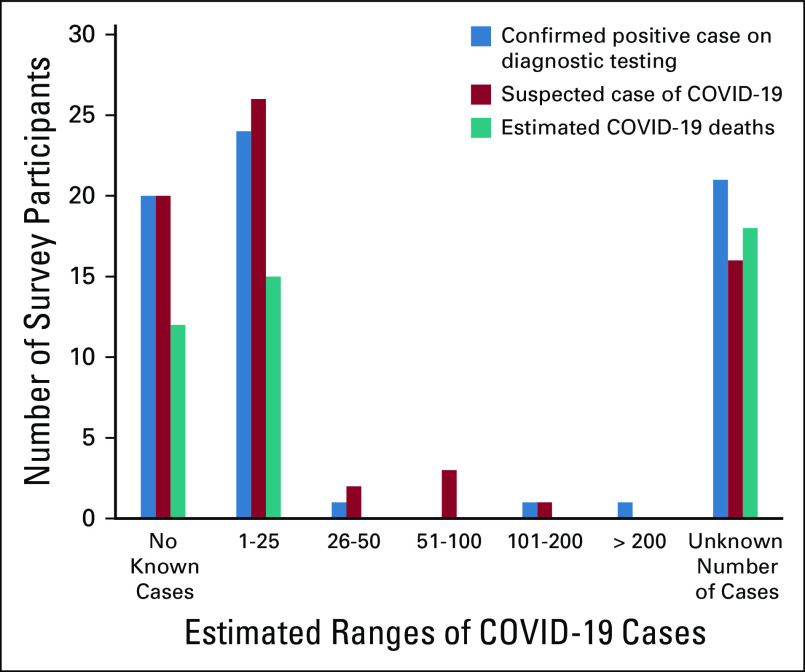
Estimated number of diagnostically confirmed and suspected COVID-19 cases and estimated deaths reported by survey participants.

Up to 30% of respondents were unable to quantify COVID-19 suspected or confirmed cases and associated deaths at their institutions. Among all patients (including those without cancer), 27% (19 of 70) reported no confirmed cases, whereas 21.4% (11 of 70) reported > 200 cases at their respective institutions. Figure 4 shows the estimated ranges of confirmed and suspected cases and deaths because of COVID-19 among patients with cancer only. Very few COVID-19 cases were confirmed or suspected among patients with cancer. A majority of participants reported ≤ 25 confirmed COVID-19 cases (44 of 68, 64%) and ≤ 5 deaths because of COVID-19 (26 of 45, 58%) among patients with cancer.

#### COVID-19–related myths and misconceptions.

The most common COVID-19–related myths and misconceptions that oncology providers confronted among their patients were that Africans were less susceptible to the virus and that COVID-19 is less infectious in the African heat (Table 6). Potentially harmful beliefs were that ingesting alcohol (16 of 70, 22.9%) or bleach (5 of 70, 7.1%) could be beneficial. Other myths and misconceptions are reported in Table 6.

**TABLE 6:**
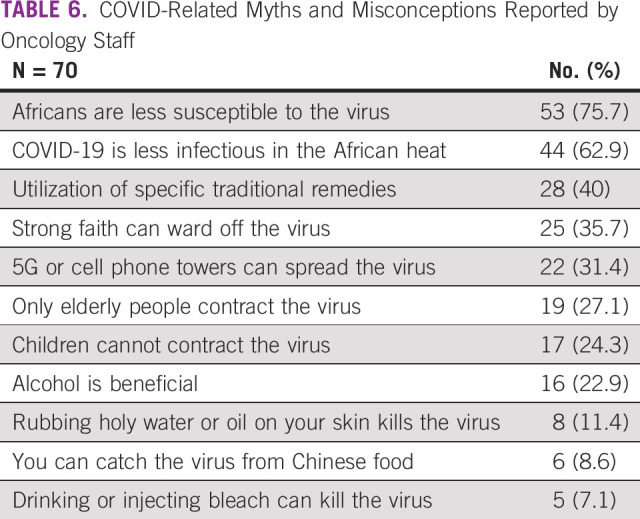
COVID-Related Myths and Misconceptions Reported by Oncology Staff

#### Discrepancies in institutional-level data.

Ten institutions had multiple respondents who provided data. There were few discrepancies in the total number of cancer cases seen per year, number of total COVID-19 cases at the institution, and specifically number of patients with cancer who have tested positive for COVID-19 or died from COVID-19 (Data Supplement). In the absence of all participants listing the name of their institution, our results are summarized based on individual data. Detailed breakdown of the data by institution where more than two participants provided discrepant data is summarized in the Data Supplement.

## DISCUSSION

In this cross-sectional study, we evaluated the scope and impact of COVID-19 and mitigating strategies on cancer care delivery in 18 countries in Africa. Consistent with a recent study,^16^ our analysis showed widespread implementation of common mitigation and suppression strategies in the institutions and countries represented in the study. The direct impact in terms of COVID-19 cases and associated deaths in patients with cancer was relatively low. However, our data highlight disruptions in care of all cancer treatment modalities, which is concerning for potentially long-term adverse sequelae for patients with cancer diagnosed and undergoing treatment during the pandemic. Not previously reported is the fear of virus transmission leading to staff shortages and decrease in patient volumes. Our data also provide insight into how the pandemic has inspired tailored innovative solutions in clinical care delivery for patients with cancer in Africa.

African countries have employed similar global mitigation and suppression strategies to flatten the curve and reduce the reproduction ratio of the virus,^23^ and several reports indicate that these governments implemented some of the most severe mitigation and suppression strategies, including state of emergency declarations and military-enforced national lockdowns.^24-26^ The data in Africa on the impact of these measures remain underestimated primarily because of limited but expanding testing capacity.^27^ Our data show few numbers of COVID-19 cases and even fewer deaths among patients with cancer. Experts are still divided over whether the low number of cases and deaths reflect successful control and social or environmental factors versus incomplete case enumeration given the low testing rates and high positivity rates in many African countries.^28,29^

Although these strategies for COVID-19 remain the underpinning of public health strategies to slow viral spread,^27,30^ our data highlight challenges in cancer care delivery for patients on the continent, which already has some of the highest case fatality rates because of cancer globally.^31^ The frequency of delays and disruptions in delivery of different cancer treatment modalities amid current mitigation and suppression strategies are concerning for long-term adverse impact on disease-free and overall cancer survival. Cancer care in sub-Saharan Africa is mostly delivered through a highly centralized model, with most cancer centers located in major cities and some patients traveling hundreds of kilometers to attend their chemotherapy infusion appointments. In the best of times, making this journey had several challenges for patients taking public transportation and making arrangements for overnight accommodation. In the current condition of strict lockdowns and public transportation bans, there are the extra transportation hurdles including obtaining travel permits needed to travel outside their homes to oncology appointments.^22,32^ These hurdles could further exacerbate delays in seeking and continuing cancer care. In Africa, where the majority of patients are diagnosed with locally advanced or metastatic disease, these disruptions in care can result in catastrophic increases in cancer-associated mortality.

Our analyses show challenges of personnel and resource shortages because of the pandemic. At baseline, oncologists in Africa have substantially higher workload compared with the global median.^33,34^ The pandemic has worsened staff shortages because of isolation and fear of the contracting the virus, particularly in settings where PPE shortages were widely reported. PPE shortages are not unique to Africa during the pandemic, as supply chains have been stressed globally. However, up to a third of participants were without the most basic access to PPE (gloves and surgical masks).

The pandemic has spurred the widespread utilization of telemedicine for care delivery.^16^ In Africa as well, our data highlight that telemedicine is being commonly used, but coupled with challenges of internet connectivity. Institutions are also increasingly using virtual tumor boards for patient discussions. The pandemic has presented an opportunity for innovation that is tailored to the healthcare systems in Africa and the needs of their patients with cancer. Similar to the results of our study, reports from Rwanda show that drones are currently being used to deliver medications to patients with cancer in an effort to avoid treatment interruptions.^35^ In Ghana and other countries, drone delivery services previously used for distribution of vaccines are now being used to collect blood and other samples from local facilities to major cities for centralized testing.^36^ This leapfrogging shows the potential driving force of a pandemic in unburdening overwhelmed medical centers focused on COVID-19 while ensuring that access to care is sustained for patients with cancer. The cost-effectiveness of these newer models is unknown but could potentially reduce cancer deaths by allowing patients with cancer to continue therapy from home where possible.

This study has several limitations. Given the anonymity of our sampling methodology, we were unable to limit multiple responses. However, the survey introduction and consent page had highlighted in bold caps that the survey was to be completed once. The sample may not be representative of all centers across the continent as there is a potential for selection bias in distributing via the AORTIC network as countries with relatively more resources allocated for oncology care may be represented in AORTIC. There is also a disproportionate representation of countries based on number of respondents who participated in the survey. The small sample size of country-level respondents, as well as differences in healthcare resources, limits the generalizability of our results to all African countries, and the data should be interpreted with caution. However, our results are consistent with a recent publication of data from 54 cancer institutions mainly from Northern Africa. The large number of participants who were unable to quantify COVID-19 cases and deaths, as well as the discrepant institutional data, suggests that staff do not have access to a centralized database with institutional data and reports of COVID-19 cases may be estimates and anecdotal, thus limiting their reliability. Furthermore, as the pandemic waxes and wanes, some of the numbers in cases and deaths might have varied based on when the survey was completed.

Our study has the key strength that the data collected provide initial critical insights into COVID-19 and its impact on cancer care delivery in Africa. Another strength is the diversity of healthcare providers represented in the study, providing a broader oncology workforce perspective. These data, especially from sub-Saharan Africa, are currently very limited but critical for formulating effective policies to ensure that patients with cancer and staff are not adversely affected by this pandemic.

In conclusion, this study shows that COVID-19 response strategies might have resulted in few direct COVID-19 cases and deaths among patients with cancer in Africa. However, substantial disruptions in all modalities of cancer treatment were reported. As data continue to expand, there is an enormous capacity to learn from the successes and failures and to rapidly change response to the pandemic in a way that ensures health equity for all patients, especially patients with cancer in Africa. It is critical to acknowledge that the death toll from COVID-19 includes not only mortality from the virus but also cancer-related deaths because of care disruption. Future research should explore the scope of COVID-19 treatment disruptions on specific cancer types and treatment regimens for cancer care delivery in Africa.
